# What’s another year? The lengthening training and career paths of scientists

**DOI:** 10.1371/journal.pone.0285550

**Published:** 2023-05-24

**Authors:** Stephanie D. Cheng

**Affiliations:** Department of Economics, Harvard University, Cambridge, MA, United States of America; Iowa State University, UNITED STATES

## Abstract

Lengthening doctorate and post-doctorate training allow science, technology, engineering, and mathematics (STEM) Ph.Ds. to persist in high-intensity academic research environments at the cost of significant lifetime earnings. Using the largest longitudinal survey of U.S. Ph.D. recipients, I construct career paths for 135,599 STEM research doctorate holders over six job types and two employment statuses. Examining Ph.D. cohorts in four major STEM fields from 1950 to the present, I find evidence that the increasingly prevalent postdoctoral position allow STEM Ph.Ds. to remain in high-intensity academic research positions, albeit not necessarily on the tenure-track. However, these research opportunities come with an approximately $3,700 deduction in annual earnings per postdoctoral year. Taken together, STEM Ph.Ds. must weigh the non-pecuniary costs of remaining in academic research with this earnings loss to determine if postdoctoral positions are a worthwhile investment.

## Introduction

The scientific community has long been concerned with the lengthening training of science, technology, engineering, and mathematics (STEM) Ph.Ds. for a shrinking number of academic tenure-track positions [1]. However, limited research has focused on tracking the career outcomes of new doctorate earners and postdoctoral researchers—especially outside of the biomedical fields [2–8]. A larger literature has focused on the transition of pre-doctorate students to graduate programs (e.g. [9–13]) or alternatively the progression of well-established scientists (e.g. [14, 15]). The little attention on post-doctorate career outcomes is further hindered by limited data, particularly as postdoctoral researchers have been poorly tracked by their institutions [16, 17]. To fill this gap in the literature, this paper systematically examines long-term trends over the course of STEM Ph.D. career paths and across multiple fields of study.

This paper draws on restricted-use data from the largest longitudinal survey of U.S. Ph.D. recipients, the National Science Foundation (NSF)’s Survey of Earned Doctorates (SED) linked to the 1993–2015 waves of the Survey of Doctorate Recipients (SDR). The SED is a census of all Ph.D. recipients from U.S. institutions administered in the year of graduation. From the SED, a nationally representative sample are followed on a biennial basis until they reach the age of 76, emigrate from the U.S., or are otherwise unable to respond. Expanding on Ginther & Kahn (2017)’s methodology for estimating postdoctoral incidence, I use the extensive survey information collected by the SED-SDR to determine each post-Ph.D. year that an individual spends any portion of the year working in six job types—postdoctoral researcher, academic tenure-track, academic non-tenure track, for-profit industry, non-profit, and government—and in two employment statuses—unemployed and out of the labor force (see Methods section and S1 Appendix for details) [18]. I compare how postdoctoral training, career paths, and job characteristics after graduation change across 1950–2013 Ph.D. cohorts.

Of particular interest is the impact of postdoctoral experience on a STEM Ph.D.’s career trajectory and lifetime earnings. I examine the transition probabilities of individuals without and with postdoctoral experience to academic tenure-track, academic non-tenure track, and industry positions. I then modify Bhuller, Mogstad, & Salvanes (2017)’s schooling regression in Eq (1) to analyze the impact of an additional year of postdoctoral training on post-Ph.D. salary paths over a thirty-year career [19].

$$\begin{array}{c} Y_{a}=\alpha_{a}+\beta_{a}P+\epsilon_{a} \end{array}$$ (1)

where in each year post-Ph.D. graduation *a*, *Y*_*a*_ gives annual real salary (in 2015 dollars) and *P* gives years of postdoctoral experience. I include fixed effects for Ph.D. field of study, graduation year, and current job type indicators. I use the yearly postdoctoral coefficient estimates to compute the average yearly postdoctoral earnings premium (or deduction) in Eq (2).

$$\begin{array}{c} \bar{\beta }=\sum_{a=0}^{30}\frac{\beta_{a}}{30} \end{array}$$ (2)

Overall, I construct a dataset consisting of 135,599 STEM Ph.D. recipients with 258,873 unique jobs. Results in the main text give the average values across all STEM fields in the SED-SDR data. Figures in the main text give results for the biological sciences, which compose 23.9% of the STEM sample and are a large driver in the overall STEM trends. S2 Appendix provides results for chemistry (10.6%), engineering (22.0%), and physics (6.3%).

## Results

**Fig 1:**
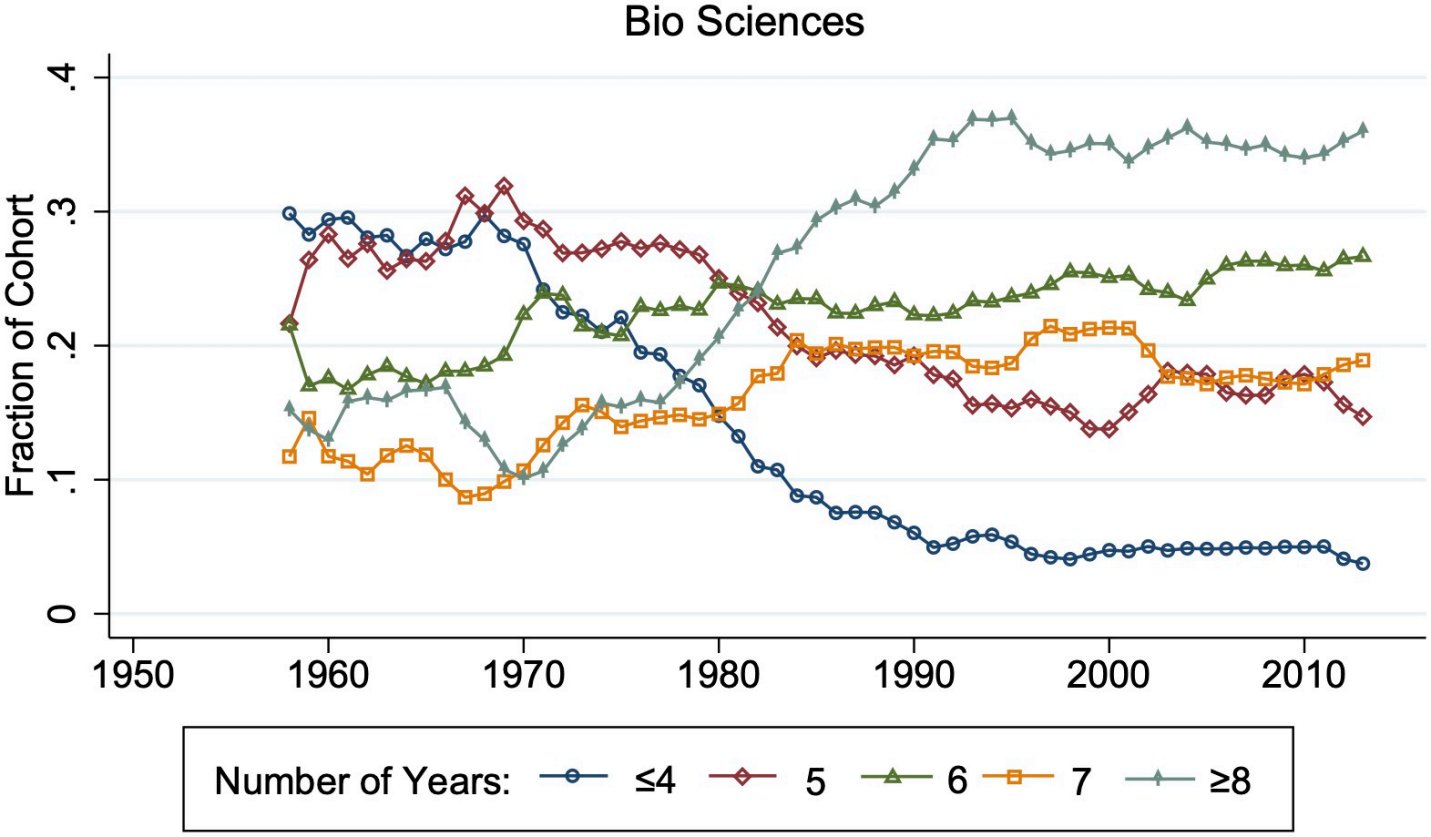
Distribution of years in graduate school by Ph.D. cohort. This graph gives the three-year moving distribution of biological science Ph.Ds.’ years spent in graduate school, defined as the time between the Bachelor’s and Ph.D. graduation year minus the number of years spent out of school during this time. Years are rounded down to the nearest integer. N ranges from {67, 1067}.

I find that the average time STEM Ph.Ds. spend in graduate school has increased from 5.8 years (s.d. = 2.1) among 1960–1980 graduating cohorts to 8.0 years (s.d. = 4.1) among 2000–2013 cohorts. This increase is not explained by individuals taking more time off between undergraduate and graduate school, which has remained steady at approximately 1.3 years (s.d. = 2.7). Rather, time in graduate school has shifted upwards: Fig 1 demonstrates for biological science Ph.Ds. that later cohorts have fewer individuals completing Ph.Ds. in under four years and more individuals completing Ph.Ds. in over eight years.

**Fig 2:**
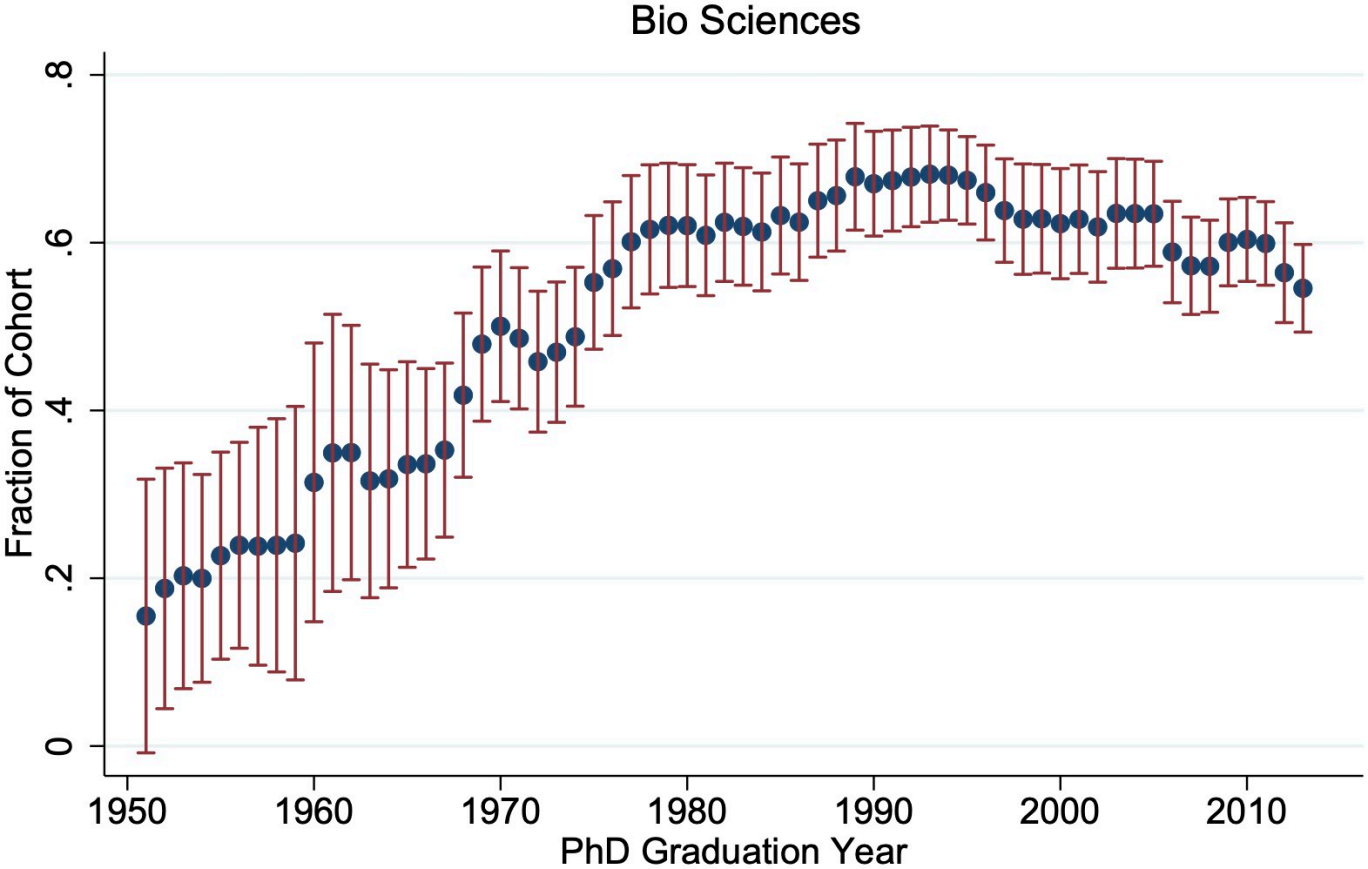
Early postdoctoral takeup by Ph.D. cohort. This graph gives the three-year moving 95% confidence intervals for the fraction of each biological science Ph.D. cohort that take on postdoctoral positions within two years of graduation. N ranges from {73, 1159}.

Despite the lengthening time in graduate school, the percent of STEM PhD graduates pursuing postdoctoral appointments grew from 28.9% of 1960–1980 cohorts to 40.2% of 2000–2013 cohorts. As shown in Fig 2, this trend is especially prevalent in the biological sciences—over 60% of 2000–2013 biological science Ph.D. graduates transition directly to postdoctoral positions, compared to only 20% of 1950’s cohorts—but fields with traditionally strong ties to industry, such as chemistry and engineering, have also seen large increases in postdoctoral takeup.

Time spent in postdoctoral positions has not varied significantly in this time period despite more individuals pursuing these positions. Conditional on any postdoctoral experience, the average time spent in postdoctoral positions across all STEM fields since 1970 is 2.7 years (s.d. = 2.3). This suggests that the purpose of postdoctoral positions has not significantly changed over time. Unlike the concurrent lengthening of graduate school, which arguably stems from requiring more time to build up a base of scientific knowledge, the rapid expansion of scientific literature in the last fifty years has not led to longer specialized training at the postdoctoral level. All together, between graduate and postdoctoral training, STEM Ph.Ds. now spend on average 9.1 years in training before their first permanent position.

**Fig 3:**
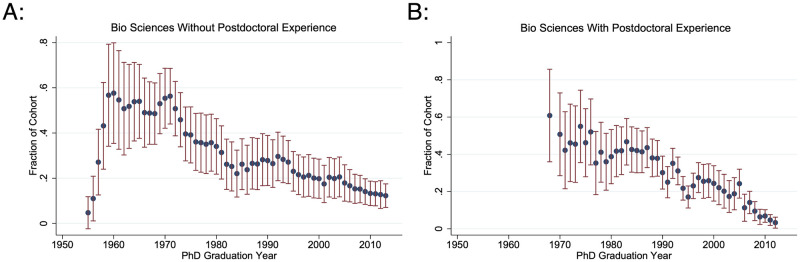
Fraction observed in an academic tenure-track position without and with postdoctoral experience by Ph.D. cohort. These graphs give the three-year moving 95% confidence intervals for the fraction of each biological science Ph.D. cohort observed in an academic tenure-track position. A: Within two years of their Ph.D. graduation without any postdoctoral experience. B: Within two years after their last postdoctoral appointment. N ranges from {54, 648}.

As doctoral training has lengthened and more STEM Ph.Ds. have pursued postdoctoral training, the probability of obtaining an academic tenure-track position has plummeted over the past fifty years. On average, only 25.2% of 2000–2013 STEM Ph.D. graduating cohorts are ever observed in a tenure-track position, compared to an average of 42.8% for 1960–1980 cohorts. Historically, postdoctoral experience improved one’s competitiveness in obtaining a tenure-track position: 40.9% of 1960–1980 STEM Ph.D. cohorts with postdoctoral experience transitioned to tenure-track positions, compared to 37.2% of those graduating in the same years with no postdoctoral experience. However, 2000–2013 graduating Ph.Ds. with postdoctoral experience are no longer more likely to obtain tenure-track positions compared to those without postdoctoral experience (Fig 3). 18.4% of STEM postdoctoral researchers who graduated between 2000–2013 transition to tenure-track jobs, compared to 21.8% directly from graduate school. Thus, there is no evidence to suggest that postdoctoral experience improves one’s job prospects on the academic tenure track.

**Fig 4:**
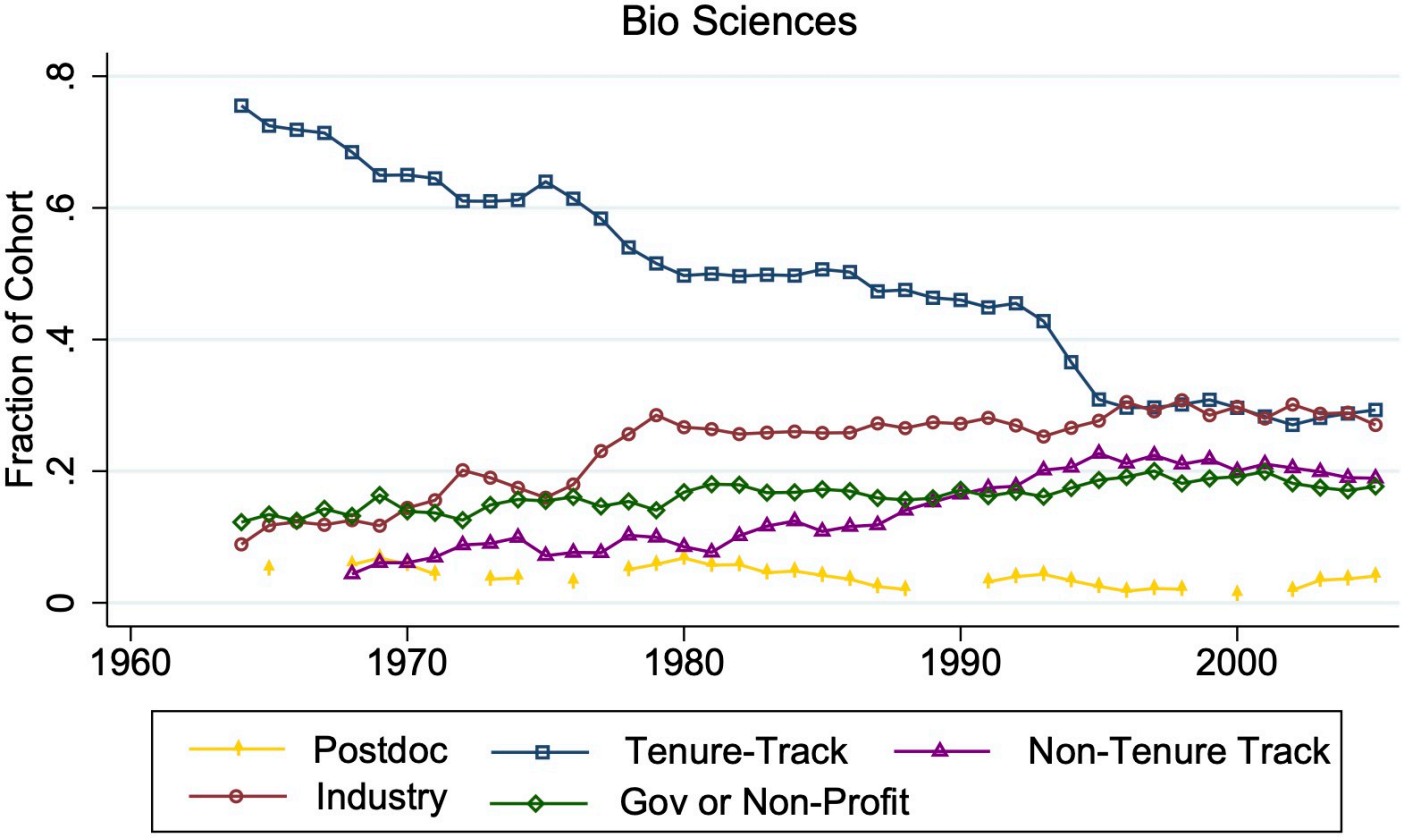
Job distributions ten years post-Ph.D. graduation. This graph gives the three-year moving fraction of each biological science Ph.D. cohort working ten years post-Ph.D. graduation in each job type. Individuals who are not working or do not have data ten years post-Ph.D. are not included. N ranges from {58, 482}.

It is now more likely that STEM Ph.Ds. work in job sectors outside of tenure-track academia (Fig 4). Although 47.5% of 1960–1980 STEM Ph.Ds. were in tenure-track academic positions ten years after their Ph.D. graduation, 2000–2013 cohorts are almost evenly distributed across tenure-track (28.1%), industry (36.0%), non-tenure track (16.6%), and government or non-profits (15.7%).

**Fig 5:**
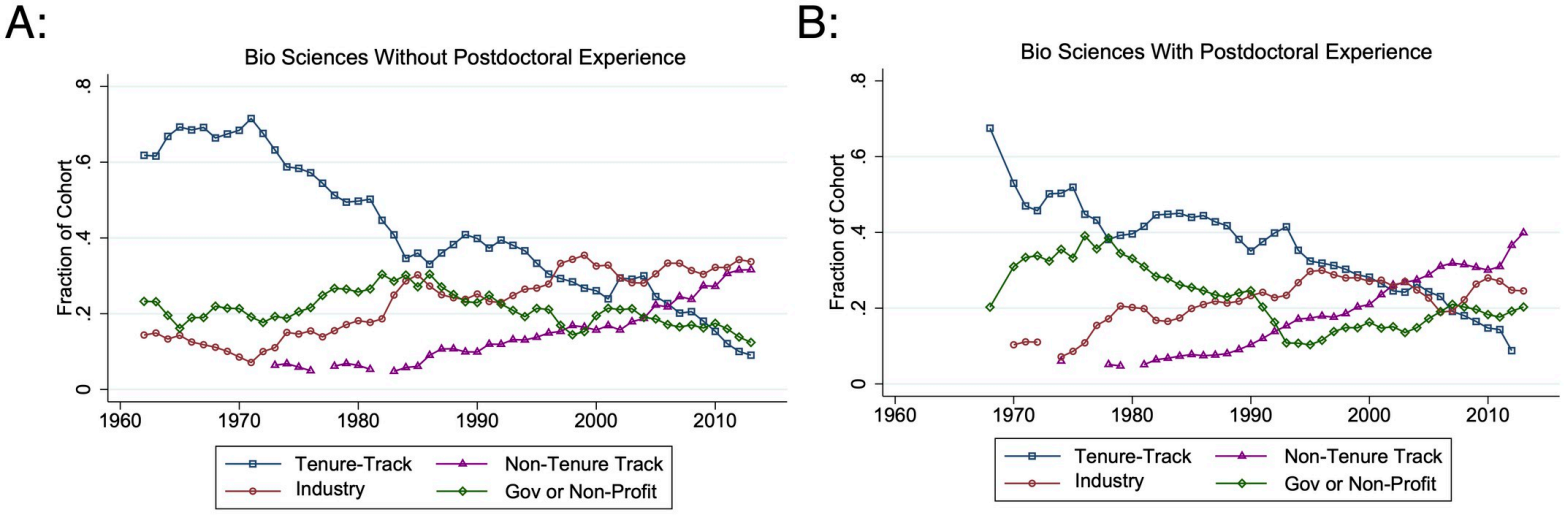
Distribution of non-postdoctoral job transitions without and with postdoctoral experience by Ph.D. cohort. These graphs give the three-year moving fraction of each biological science Ph.D. cohort who transition into each non-postdoc job type. A: Within two years of their graduation with no postdoctoral experience. B: Within two years after their last postdoctoral appointment. N ranges from {52, 436}.

Moving away from the tenure track and into “alternative” job sectors occurs for both individuals transitioning directly from graduate school and those who transition from postdoctoral appointments. Compared to 17.9% of 1960–1980 cohorts, 22.8% of 2000–2013 cohorts transition to for-profit industry directly from graduate school. Especially among postdoctoral researchers, academic non-tenure track positions have become increasingly popular. Among 2000–2013 STEM Ph.D. cohorts, 8.7% of new graduates and 23.9% of postdoctoral researchers transitioned into non-tenure track jobs (Fig 5).

As a larger percent of postdoctoral researchers transition to academic non-tenure track jobs than new Ph.D. graduates, this may indicate that individuals with a higher preference for academic jobs are selecting into postdoctoral positions, regardless of the possibility of tenure. Previous literature has documented researchers’ willingness to trade off salary for greater research time [20–24]. Consistent with the literature, many STEM Ph.Ds. pursue academic positions that have higher research activities but lower salaries compared to industry positions. When asked the job activity on which they spend the most time, only 2.3% of STEM Ph.Ds. at for-profit industry jobs spend the most time on basic research but have the highest average salary at $127,469. Comparatively, 21.8% of tenure-track positions and 23.5% of non-tenure track positions spend the most time on basic research and have an average salary of $99,500 and $71,680 respectively. Of all job types, postdoctoral positions perform the most basic research and have the lowest salary: 42.0% of all postdoctoral positions spend the most time on basic research at an average salary of $50,396.

Given the limited number of academic tenure-track positions, a postdoctoral appointment may allow STEM Ph.Ds. the flexibility to wait for high-research permanent positions. The majority of individuals in non-postdoctoral jobs are never observed switching to any other job type, compared to only 13.3% of postdoctoral researchers remaining in the position throughout their observed career path. Because individuals do not typically move between permanent job sectors, an individual who leaves academic research for another permanent job type is unlikely to ever return. By remaining in a transitory state like a postdoctoral position, STEM Ph.Ds. have more flexibility to move to any of the permanent job types.

**Fig 6:**
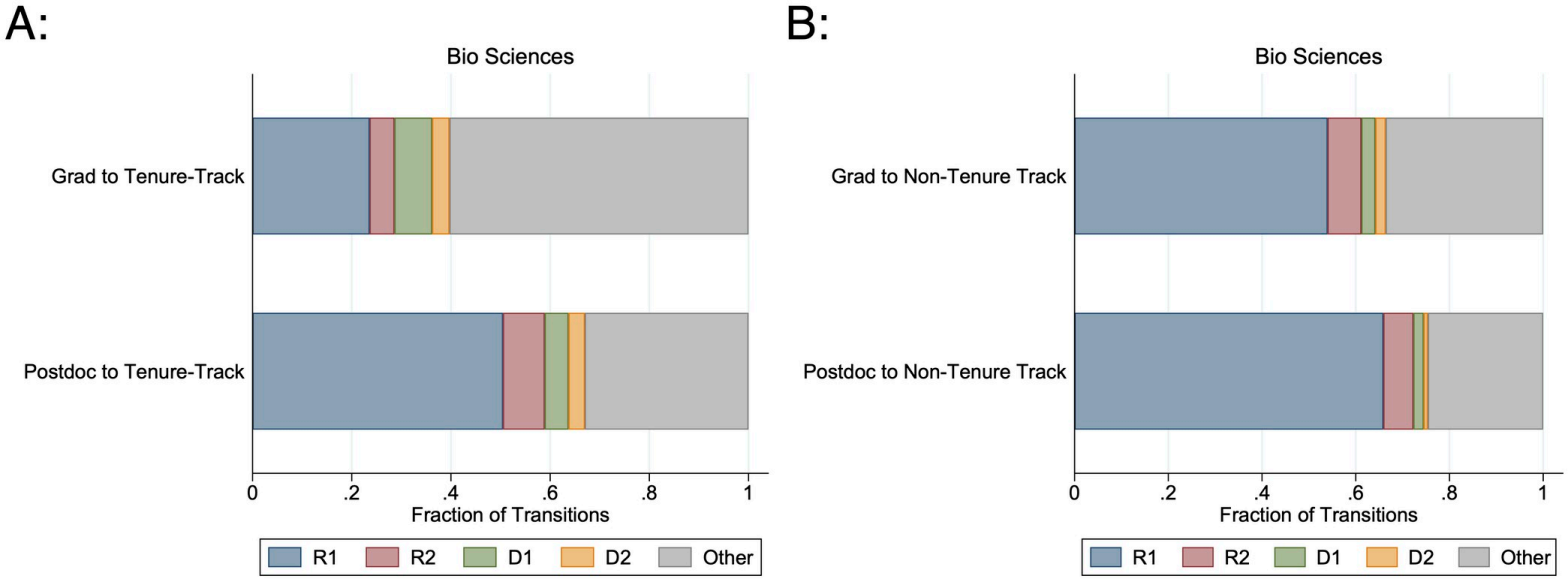
Institutional carnegie classifications for academic transitions without and with postdoctoral experience. This graph gives the distribution of known Institutional Carnegie Classification for biological science Ph.Ds.’ academic transitions. A: Transitions to tenure-track positions. B: Transitions to non-tenure track positions. “Grad” transitions are within two years of their Ph.D. graduation with no postdoctoral experience. “Postdoc” transitions are within two years after their last postdoctoral appointment. Institutional prestige is highest among R1 (“very high research”) institutions, followed by R2 (“high research”), D1 (“doctoral I”), D2 (“doctoral II”), and Other (no available classification). N ranges from {654, 4722}.

STEM postdoctoral researchers also spend more time at research-intensive universities than individuals who transition directly from graduate school (Fig 6). 78.7% of first postdoctoral positions are at Carnegie-Classified R1 “very high research activity” institutions. Although postdoctoral researchers are not more likely to transition to any tenure-track position than new graduates, those who are able to obtain a permanent academic position are more likely to be at very high research activity universities. Among new graduates transitioning to academic positions, 29.5% of tenure-track transitions and 49.5% of non-tenure track transitions are at R1 universities. Among postdoctoral researchers transitioning to academic positions, 51.2% of tenure-track transitions and 67.9% of non-tenure track transitions are at R1 universities. This indicates that individuals pursuing postdoctoral positions are not of lower ability than those who move directly into permanent positions; rather, they may have a higher threshold in the level of research activity they would accept for a permanent position.

**Fig 7:**
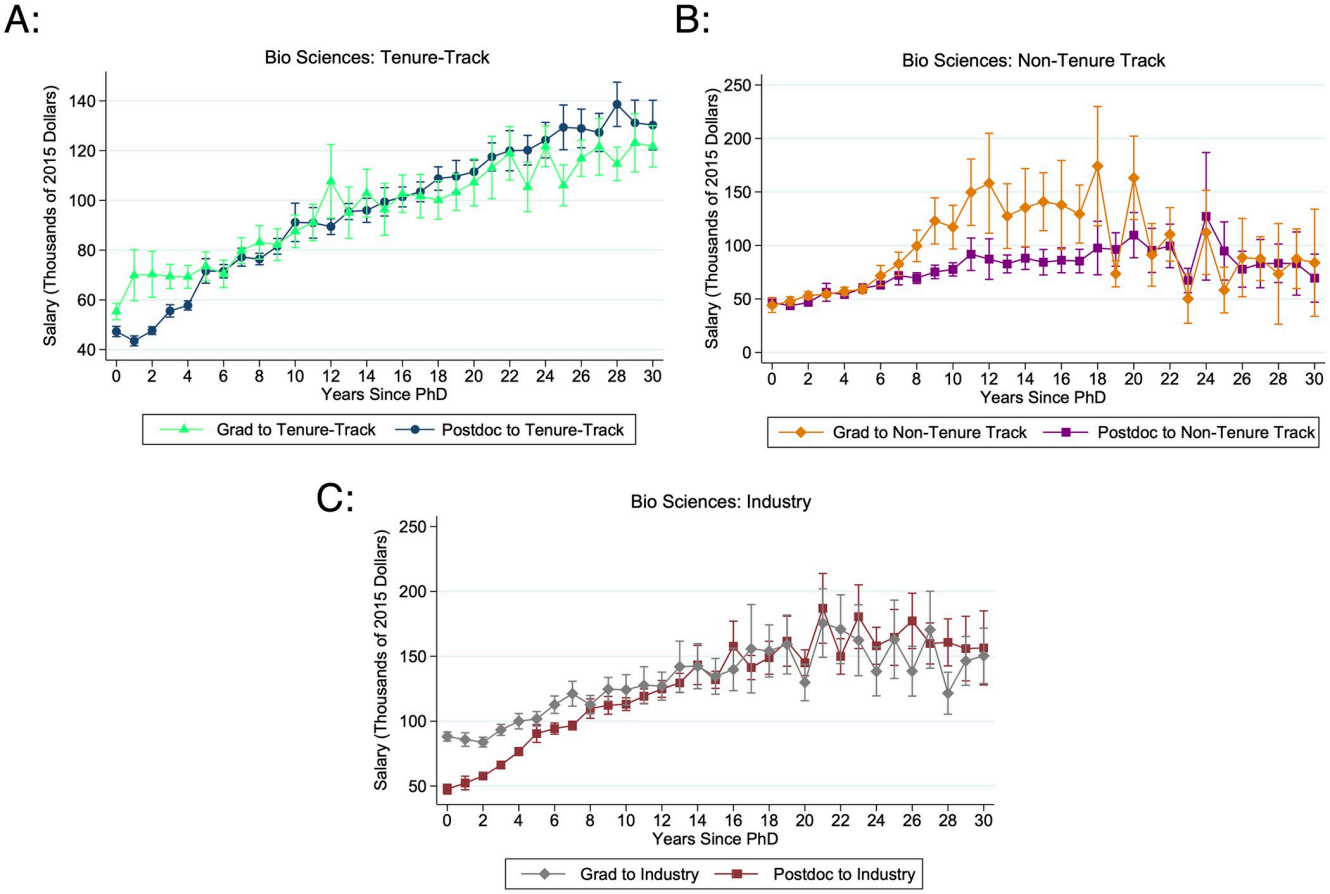
Average salary in each year since Ph.D. graduation by postdoctoral path. These graphs give the average salary over the first thirty years after Ph.D. graduation for biological science Ph.Ds. without and with any postdoctoral experience. A: Tenure-track jobs. B: Non-tenure track jobs. C: Industry jobs. N ranges from {71, 3023}.

However, waiting for this academic research position comes at a significant cost to postdoctoral researchers’ lifetime earnings. Although postdoctoral experience provides a small improvement in starting salary and growth over time, this does not offset the significant losses from taking a low-paying position early in the career. Over the first thirty years after their Ph.D. graduation, having any postdoctoral experience is associated with a decrease of $5,333 (tenure-track), $10,626 (non-tenure track), and $13,549 (industry) in average yearly earnings (Fig 7).

**Fig 8:**
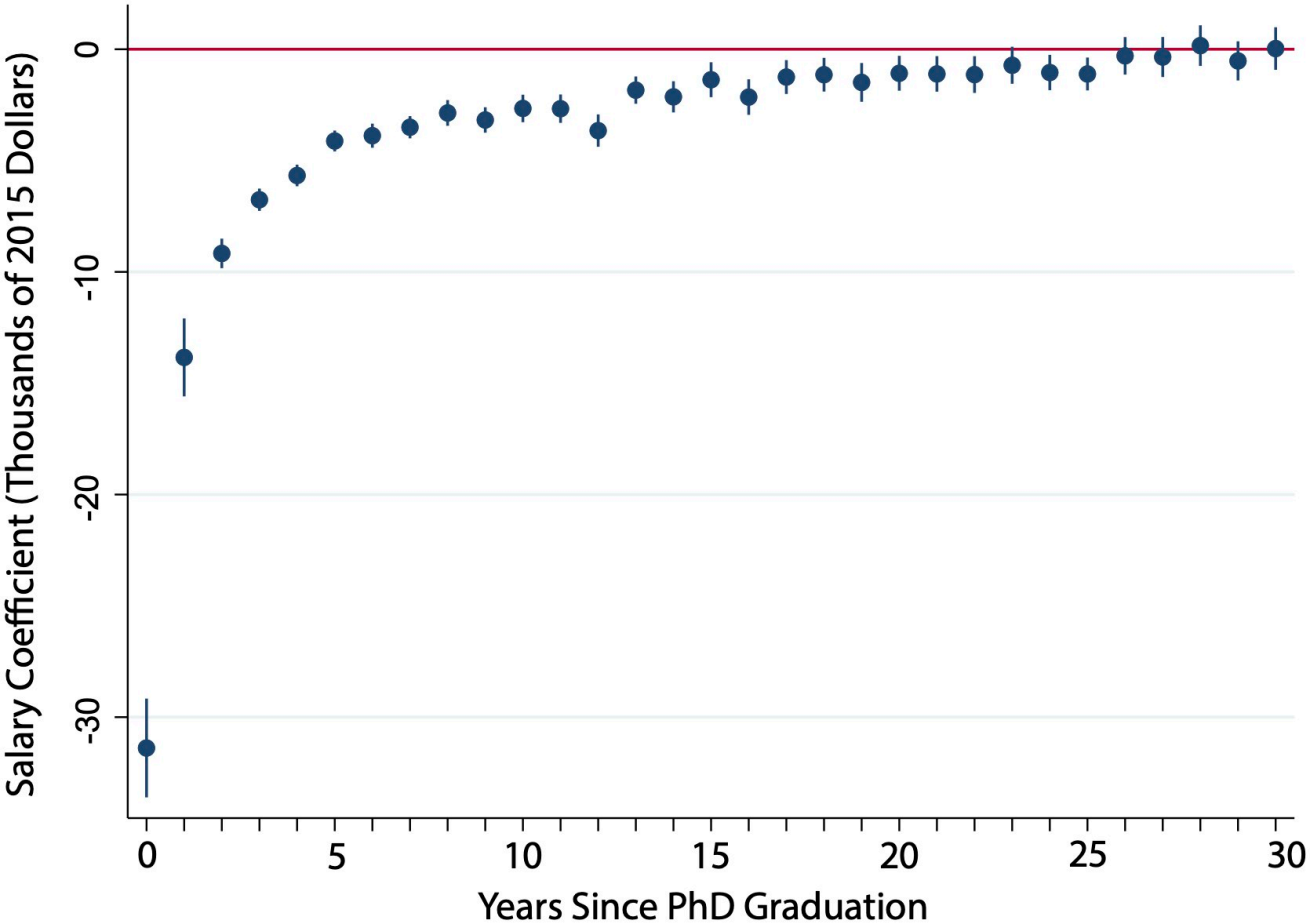
Salary regression coefficients on postdoctoral experience in each year post-Ph.D. graduation. This graph gives the salary regression coefficients on years of postdoctoral experience for the first thirty since Ph.D. graduation. Regression includes fixed effects for Ph.D. field of study, graduation year, and job type (tenure-track, non-tenure track, or industry) and is performed on all STEM fields. N ranges from {4595, 12557}.

The regression in Eq (1) measures the impact of each additional year of postdoctoral experience on salary. As shown in Fig 8, the first few post-Ph.D. years show a large negative relationship due to the salary gap between postdoctoral appointments and permanent positions. This gap closes as postdoctoral researchers move into permanent positions, but the additional training does not improve their salaries enough to overcome this early loss. As given in Eq (2), the average of these yearly coefficients can be interpreted as the postdoctoral deduction in mean lifetime earnings. Rather than provide an education premium, each additional year of postdoctoral experience reduces average annual earnings by $3,730.

## Discussion

Across STEM fields, I find that training time has increased significantly since the 1960’s. At the same time, postdoctoral positions are becoming more commonplace even in fields with strong industry ties like engineering. However, the probability of ever obtaining a tenure-track position has declined in the same period, ranging from an approximately 14 percentage point decline in chemistry to as much as 36 percentage points in the biological sciences. In recent years, having postdoctoral experience does not significantly improve one’s chances of obtaining a tenure-track job: although nearly 41 percent of 1960–1980 STEM Ph.D. cohorts with postdoctoral experience transitioned to tenure-track positions, fewer than 20 percent of both new graduates and postdoctoral researchers from 2010–2013 cohorts transition to these positions.

Rather, I find evidence that postdoctoral positions allow STEM Ph.Ds. to stay longer in high-intensity academic research jobs—but not necessarily on the tenure track. Compared to permanent job types, in which individuals are unlikely to move, temporary postdoctoral appointments provide STEM Ph.Ds. more flexibility to transition to a new job type. Although postdoctoral researchers are not more likely to enter a tenure-track position than new Ph.D. graduates, they are 15 percentage points more likely to transition to non-tenure track positions with high levels of basic research activity. Conditional on transitioning to a permanent academic position, postdoctoral researchers are approximately 20 percentage points more likely to take a position at a Carnegie-Classified “very high research activity” institution compared to those who transition directly from graduate school. This indicates that those pursuing postdoctoral positions are not of lower research ability than those transitioning directly from graduate school; rather, they may have a higher preference for academic research jobs. With approximately 79% of postdoctoral positions at very high research activity universities, postdoctoral positions allow individuals to remain in these high-intensity research environments longer.

However, this research opportunity comes at a significant cost in lifetime earnings. Although postdoctoral researchers transition into permanent positions with equal or higher starting salaries as individuals who transition directly from graduate school, their thirty-year salary growth is not large enough to compensate for the low postdoctoral pay early in their careers. Rather than provide an education premium, each additional postdoctoral year is associated with a $3,730 decrease in undiscounted average annual earnings. This negative salary effect must thus be weighed against the non-pecuniary benefits of postdoctoral positions (e.g. the preference for high-intensity academic research) to determine whether it is a worthwhile investment.

Despite the longer training and declining probability of ever obtaining a tenure-track position, recent STEM Ph.D. cohorts are more likely to pursue postdoctoral positions than their predecessors. Although these postdoctoral positions do not improve lifetime earnings, they allow STEM Ph.Ds. to remain in preferred high-intensity academic research positions. One potential test for this mechanism is whether the decision to pursue a postdoctoral appointment changes in response to hypothetical or actual shocks to the availability of positions at very high research activity universities. This can be explored in future research using hypothetical choice surveys or labor market shocks. The postdoctoral deduction in lifetime earnings also begs the question of which types of STEM Ph.Ds. are able to afford this cost to pursue the non-pecuniary benefits. For example, an extension of the methods in this paper finds that women raising children are less likely to persist on the academic tenure track due to longer working hours during these crucial transitional periods [25]. If these time and financial constraints limit who can pursue postdoctoral positions and thus remain in high-intensity academic research environments, it may explain diversity gaps in the STEM pipeline. Understanding why individuals choose to pursue postdoctoral positions allows policymakers to better understand mechanisms driving the STEM labor market. Continuing this work can identify improvements for STEM training programs, encourage persistence in scientific research across a diverse workforce, and prepare STEM Ph.Ds. for the wide range of career paths they can pursue today.

## Methods

This paper constructs career paths for all individuals in the 1993–2015 SDR survey waves. Job types in each year are identified based on the SDR’s extensive questioning of a respondent’s job characteristics, job tenure, and retrospectives of previous positions. Sampling weights for each respondent are used to account for differential sampling rates and adjustments for unknown eligibility, non-response, and demographic extrapolation to the population distribution. S1 Appendix details this career path construction and gives a hypothetical example using this methodology. This project was submitted for written IRB approval at Harvard University under ID IRB18–0447 and was deemed “not human research” based on its use of existing survey data collected by a third party (NSF).

Note that one limitation of the SED-SDR is that the surveys report limited information on research ability proxies, a potentially helpful control in separating an individual Ph.D.’s ability to obtain an academic tenure-track job from personal preferences for such jobs. A few survey waves (1995, 2001, 2003, and 2008) ask respondents about their five-year publication and patent rates; this question has since been discontinued. No question asks about cumulative number of publications or number of patents. Thus, I am limited to information at the academic institution level for an individual respondent’s ability proxy. I use the Carnegie Classifications system—which groups universities based on their research expenditures and number of doctorate degrees earned—as a measure of educational prestige. The SED-SDR data provides Carnegie Classifications for the academic institutions from which an individual receives their Bachelor’s, Master’s, and Doctorate degrees. If an individual works at an academic institution after their Ph.D. graduation, I further merge on the institution’s Carnegie Classification at the time of employment. I focus on the likelihood that a STEM Ph.D. or postdoctoral researcher transitions to a R1 or “very high research activity” institution, which is defined as having at least $40 million in federal research support and awarding at least fifty doctoral degrees per year (e.g. Harvard University, Stony Brook University).

## Supporting information

S1 Appendix Career paths construction. Details the methodology used to identify a SED-SDR individual’s career path.
(ZIP)

S2 Appendix Trends across STEM fields. Provides figures for three other large STEM fields: chemistry, engineering, and physics.
(PDF)
