# Supplementary material for: What’s another year? The lengthening training and career paths of scientists
Source: PLoS One. 2023 May 24;18(5):e0285550. doi: 10.1371/journal.pone.0285550 (PMC10208458; doi:10.1371/journal.pone.0285550)
Supplement: S1 Appendix — Details the methodology used to identify a SED-SDR individual’s career path. (ZIP) [file pone.0285550.s001.zip › S1_Appendix.pdf]

What's Another Year? S1 Appendix

Stephanie D. Cheng[1¤*]

1 Department of Economics, Harvard University, Cambridge, MA, USA

¤Current Address: Edgeworth Economics, Washington, DC, USA
* scheng@edgewortheconomics.com

Career Paths Construction

This appendix details the methodology used to identify a SED-SDR individual's career paths across six job types and two employment statuses. To illustrate this methodology, I use an example Ph.D. whose true career path is given in Table 1. Based on this true path, the individual fills out the job-related variables from each SED or SDR survey in Table 2. Note that this data has been constructed for example purposes and does not represent an actual individual in the SED-SDR data.

I start by identifying all individuals covered by the 1993-2015 SDR, matching to their SED responses using the variable *refid,* and using their first weight observation *wtsurvy.* For demographics that don't vary over time – race, gender, birth date, birthplace, native US citizenship, educational attainment prior to the Ph.D. (including years out of school), Ph.D. field of study, Ph.D. institution, and Ph.D. graduation year – I consider the individuals' SED responses to be the definitive source for these variables. I calculate the number of years each individual spends in graduate school by taking the difference between the year an individual receives their Ph.D. and the year they receive their Bachelor's degree, subtracting any time they spend out of school.

I identify six possible principal job types individuals can hold:

- **Postdoctoral Researcher (PD):** In the SED, the individual's postgraduation plans (given by the variable *pdocplan*) are a postdoctoral fellowship, a postdoctoral research associateship, a traineeship, or a clinical residency internship. In the SDR, the indicator for a postdoctoral principal job, *pdix*, equals one; alternatively, in the 1995 or 2006 SDR, the individual identifies this time period as a postdoctoral position through the retrospective questions on postdoctoral history (given by postdoctoral starting and ending years, *pd*syr* and *pd*eyr).*

- **Academic Tenure-Track (TT):** In the SED, the individual's postgraduation plan is not a postdoctoral position (as defined above) but is employment in a U.S. 4-year college or university, medical school, research institute, or university hospital. In the SDR, the individual is not in a postdoctoral position but is either tenured or on the tenure track (as given by the variables *facten* and *tensta*).

- **Academic Non-Tenure Track (NT):** In the SED, the individual's postgraduation plan is not a postdoctoral or tenure-track academic (as defined above) but is employment in a U.S. community college, or U.S K-12. In the SDR, the individual is not in a postdoctoral or tenure-track academic position but is employed in an educational institution (as given by the employment sector variable *emsecdt*).

S1 Table 1. Example Individual's True Career Path.

refyr (1)	PD (2)	TT (3)	NT (4)	ID (5)	NP (6)	GV (7)	UN (8)	NL (9)
1990	X							
1991	Y							
1992	Y							
1993	Y							
1994		X						
1995		X						
1996		X						
1997		X						
1998		X						
1999			X					
2000			X		X			
2001					X			
2002				X				
2003				X				
2004				Y				
2005				Y				
2006				Y				
2007							X	
2008						X		
2009						Y		
2010						Y		
2011						Y		
2012						Y		X
2013								X
2014						X		
2015						X		

This table gives the true career path of a constructed SDR individual. Column 1 gives the reference year, *refyr*. Columns 2-9 give job types and employment statuses abbreviated as postdoctoral researcher (PD), academic tenure-track (TT), academic non-tenure track (NT), for-profit industry (ID), non-profit (NP), government (GV), unemployed (UN), and not in labor force (NL). A marked box denotes employment in that job type or employment status in that year; if an individual switches jobs but remains in the same job type, different jobs are denoted by switching the markings (X, Y, etc.). For example, the individual switches from one postdoctoral position to another in 1991, so the first postdoctoral job is denoted by X and the second is denoted by Y.

S1 Table 2. Example Individual's Responses to SED/SDR.

refyr	phdcy	pdocstat	pdocplan	strtyr	pdix	lfstat	emsecdt	facten	tensta	emsmi	lwyr	pd1syr	pd1eyr	pd2syr	pd2eyr
**(1)**	**(2)**	**(3)**	**(4)**	**(5)**	**(6)**	**(7)**	**(8)**	**(9)**	**(10)**	**(11)**	**(12)**	**(13)**	**(14)**	**(15)**	**(16)**
1990	1990	2	0												
1993	1990			1991	1	1	11	4	5						
1995	1990			1994	0	1	11	1	4	4		1991	1993	1990	1990
1997	1990			1994	0	1	11	1	4	1					
1999	1991			1999	0	1	11	4	5	4					
2001	1990			2000	0	1	23			4					
2003	1990			2002	0	1	22			4					
2006	1990			2004	0	1	21			3		1991	1993	1990	1990
2008	1990			2008	0	1	32			4					
2010	1990			2008	0	1	32			2					
2013	1990				0	3					2012				
2015	1990			2014	0	1	32								

This table gives the constructed SDR individual's responses to the SED in 1990 and the 1993-2015 SDR waves, based on their true career path in Table 1. Column 1 gives the reference year for the survey, *refyr*. Column 2 reports the Ph.D. graduation calendar year, *phdcy*; note that there is a typo in the 1999 SDR response. Columns 3-4 gives the individual's post-graduation status, *pdocstat*, and post-graduation planned employment, *pdocplan*, reported in the SED. Column 5 gives the starting year, *strtyr*, for the reported principal job. Column 6 is an indicator for whether the principal job is a postdoctoral position, *pdix*. Column 7 gives the labor force status, *lfstat*. Column 8 gives the employment sector, *emsecdt*. Columns 9-10 describe the faculty rank, *facten*, and tenure status, *tensta*, for employment in academic institutions. Column 11 describes whether the individual held the same job and/or employer during the last survey, *emsmi*. Column 12 gives the last year worked if unemployed or out of the labor force, *lwyr*. Columns 13-16 give retrospective start and end dates for the two most recent postdoctoral positions, *pd1syr-pd2eyr*; in this example individual, they did not have a third postdoctoral position, so *pd3syr* and *pd3eyr* are empty for all surveys.

- **Industry (ID):** For both the SED and SDR, the individual is employed in the for-profit industry sector, for-profit business sector, or is self-employed.
- **Non-Profit (NP):** In the SED, the individual's postgraduation plan is a not-for-profit organization or international organization such as UN, UNESCO, or WHO. In the SDR, the individual is employed in a non-profit sector.
- **Government (GV):**In the SED, the individual's postgraduation plan is employment at a foreign government, U.S. federal government, U.S. state government, or U.S. local government. In the SDR, the individual is employed in the government sector.

I also examine if individuals are not employed and hold the following non-employed statuses:

- **Unemployed (UN):** There is no information on unemployment in the SED. In the SDR, an individual's labor force status is unemployed (as given by the variable *lfstat).*
- **Not in Labor Force (NL):** In the SED, the individual's postgraduation status is not seeking employment (including being a housewife, writing a book, or no employment). In the SDR, the individual's labor force status is not in the labor force.

From the SED, I identify STEM Ph.Ds.' immediate post-graduation status using the variables *pdocstat*. Individuals are considered to be in a particular job type the year of their graduation if they indicated they are returning to employment, have a signed contract, or are in negotiations for that job type. From the SDR, I utilize variables on their current job (e.g. *pdix* - indicator for postdoctoral principal job, *facten* - faculty rank and tenure status, *tensta* - tenure status, *emsecdt* - employer sector, and *lfstat* - labor force status), comparison to their previous job (e.g. *emsmi* asks if individual holds the same employer and/or same job as the last SDR survey, typically two to three years earlier), and retrospective postdoctoral experience asked of respondents in 1995 and 2006 (e.g. *pd*syr* and *pd*eyr* give start and end years for the three most recent postdoctoral appointments). Because some variables impart more information about one's job type than others, I use the following hierarchy to fill in indicators for each job type in each year from 1945-2015:

1. **New job:** Individual is starting a new job (given by start date) in that year. In the case of unemployed or out of labor force, the last year worked was the previous year.
2. **Postdoctoral retrospective:** Individual stated they were in a postdoctoral position in the retrospective 1995 and 2006 data, as given by the postdoctoral start and end dates. Fill indicators for all years between the start and end years.
3. **Current job:** Individual is currently in this job type; fill indicators for all years up through starting year. In the case of unemployed or out of labor force, fill indicators for all years just up to the year last worked.
4. **In same job type last survey:** Individual states they were either 1) in the same job and same employer, 2) in the same job but had a different employer, or 3) had the same employer but different job as the last survey. Denote these as case 4, case 4.1, and case 4.2 respectively. Fill indicators for current job type up to last survey year.

5. **Expected post-graduation job:** Fill in job type for an individual's graduation year from their expected post-graduation job type, as given by the SED.

6. **No other information, expected transition:** If steps 1-5 have not given any information on an individual's job type in a particular year but have given information in the previous year, assume that individuals were in the same job type as the year had information.

7. **No information expected:** For years before completing the Ph.D. and after the last year surveyed, the individual contributes no further information about their job type, so replace indicators with missing.

The example individual's indicators are given in Table 3. I consider the highest step in the hierarchy as the most accurate representation of whether an individual was in that job type in that year.

To estimate the number of years an individual is in a particular job type, I count one year for each year an indicator's most definitive step is steps 1-5 and a half year for each year an indicator's most definitive step is step 6. Transitions are defined by the new job type within two years of the last year spent in a different job type. As shown in Table 3, the example individual is considered to have spent four years in a postdoctoral position, four and a half years in academic tenure-track, one year in non-tenure track, two years in non-profit, five and a half years in industry, two years not in labor force, and five and a half years in government. They have switched from postdoctoral to tenure track, tenure track to non-tenure track, non-tenure track to non-profit, non-profit to industry, industry to government, and not in labor force to government.

This methodology is able to capture the majority of the true career path; however, the example also illustrates limitations when individuals switch principal jobs between survey years or have employment gaps for a year or less. The 1999-2000 non-tenure track and the 2009-2012 government positions are underestimated, as the individual switched to a different job type in a non-survey year. The 2007 unemployment gap is missed due to being in a non-survey year. The 2004-2006 for-profit job is overestimated due to a lack of job type information in 2007. Since transitions are defined by the last time an individual is observed in a job type, this methodology also misses the transition from government to not in labor force (as the individual returns to government later on).

S1 Table 3. Example Constructed Career Path.

**refyr**	**PD**	**TT**	**NT**	**ID**	**NP**	**GV**	**UN**	**NL**
**(1)**	**(2)**	**(3)**	**(4)**	**(5)**	**(6)**	**(7)**	**(8)**	**(9)**
1990	5,**2**							
1991	**1**,2							
1992	3,**2**							
1993	3,**2**							
1994		**1**						
1995		**3**,4						
1996		**3**,4						
1997		**3**,4						
1998		**6**						
1999			**1**					
2000			[ ]		**1**			
2001					**3**			
2002				**1**				
2003				**3**,4.1				
2004				**1**,4.1				
2005				**3**,4.1				
2006				**3**,4.1				
2007				[**6**]			[ ]	
2008						**1**,4.2		
2009						**3**,4.2		
2010						**3**,4.2		
2011						**6**		
2012						[ ]		**1**
2013								**3**
2014						**1**		
2015						**3**		

This table gives the constructed career path based off survey responses in Table 2. Column 1 gives the reference year, *refyr*. Columns 2-9 give job types and employment statuses abbreviated as postdoctoral researcher (PD), academic tenure-track (TT), academic non-tenure track (NT), for-profit industry (ID), non-profit (NP), government (GV), unemployed (UN), and not in labor force (NL). Boxes are marked with the steps of the hierarchy that the year satisfies: 1 denotes a new job; 2 denotes a postdoctoral position given by the retrospective module; 3 denotes a current job reaching back to its starting year; 4 denotes the same job and employer as the previous wave; 4.1 denotes the same job but different employer as the previous wave; 4.2 denotes the same employer but different job as the previous wave; 5 denotes the SED post-graduation plans; and 6 denotes an expected transition. The smallest number in each cell is bolded and used as the most accurate representation of whether the individual was in that job type in that year. Brackets denote differences from the true career path given in Table 1.
