# Supplementary material for: What’s another year? The lengthening training and career paths of scientists
Source: PLoS One. 2023 May 24;18(5):e0285550. doi: 10.1371/journal.pone.0285550 (PMC10208458; doi:10.1371/journal.pone.0285550)
Supplement: S2 Appendix — Provides figures for three other large STEM fields: chemistry, engineering, and physics. (PDF) [file pone.0285550.s002.pdf]

What's Another Year? S2 Appendix

Stephanie D. Cheng[1¤*]

1 Department of Economics, Harvard University, Cambridge, MA, USA
¤Current Address: Edgeworth Economics, Washington, DC, USA
* scheng@edgewortheconomics.com

Trends Across STEM Fields

Figures in the main text show results for the biological sciences, because this represents the largest STEM field by number of Ph.D. graduates (23.9%). In recognition that less research has been done on other STEM fields that may be of interest to specialty groups, this appendix provides the results for three additional large fields: chemistry (10.6%), engineering, (22.0%) and physics (6.3%). Note that for disclosure purposes, graphs are limited to cohorts with at least fifty individuals and cells with at least five individuals.

A:

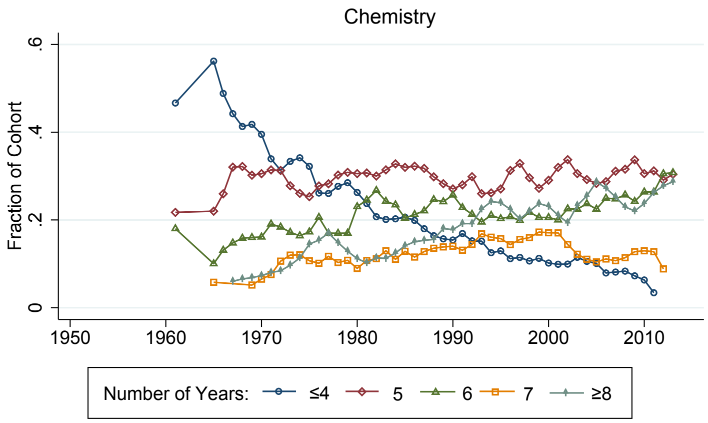

B:

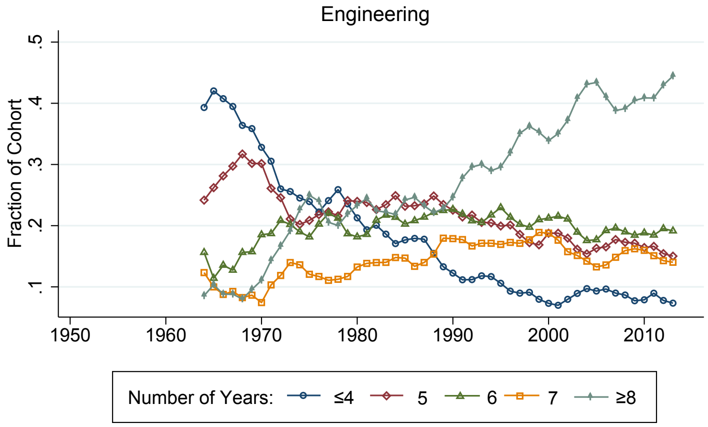

C:

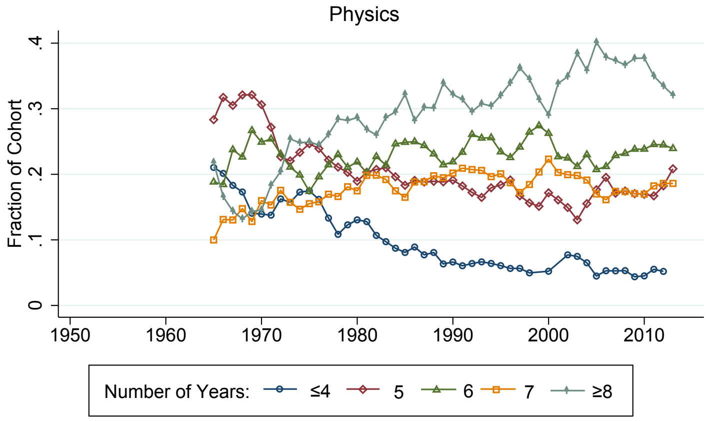

S2 Fig. 1. Distribution of Years in Graduate School by Ph.D. Cohort for Additional Fields. These graphs give the three-year moving distribution of Ph.D.s' years spent in graduate school, defined as the time between the Bachelor's and Ph.D. graduation year minus the number of years spent out of school during this time for A: Chemistry, B: Engineering, and C: Physics. Years are rounded down to the nearest integer. N ranges from {50, 255} for chemistry, {51, 923} for engineering, and {53, 249} for physics.

A:

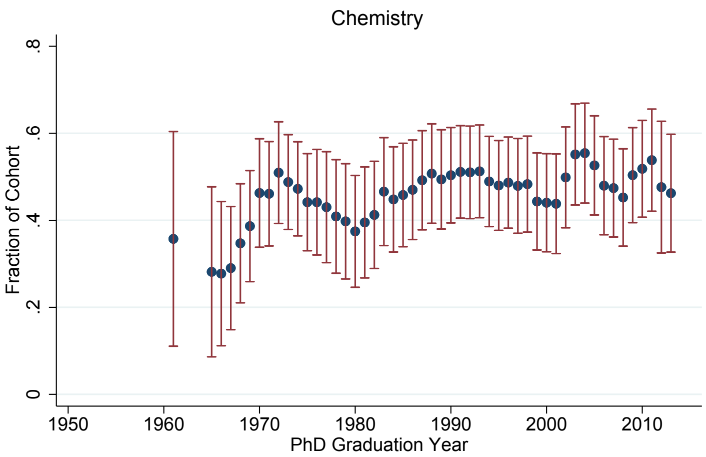

B:

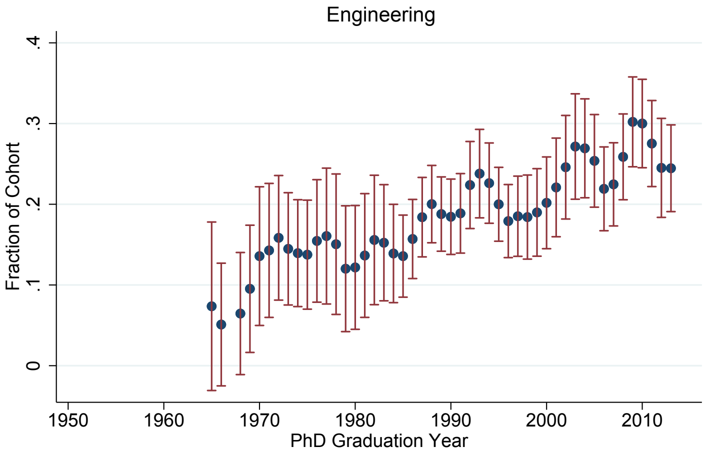

C:

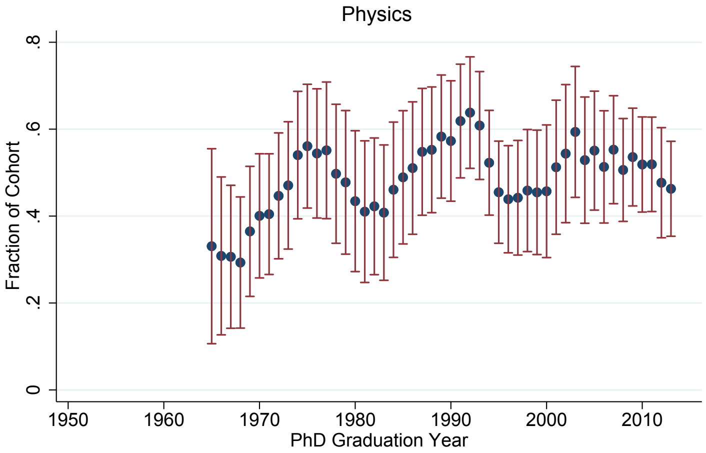

S2 Fig. 2. Early Postdoctoral Takeup by Ph.D. Cohort for Additional Fields. These graphs give the three-year moving 95% confidence intervals for the fraction of each Ph.D. cohort that take on postdoctoral positions within two years of graduation for A: Chemistry, B: Engineering, and C: Physics. N ranges from {52, 296} for chemistry, {82, 1023} for engineering, and {57, 266} for physics.

A:

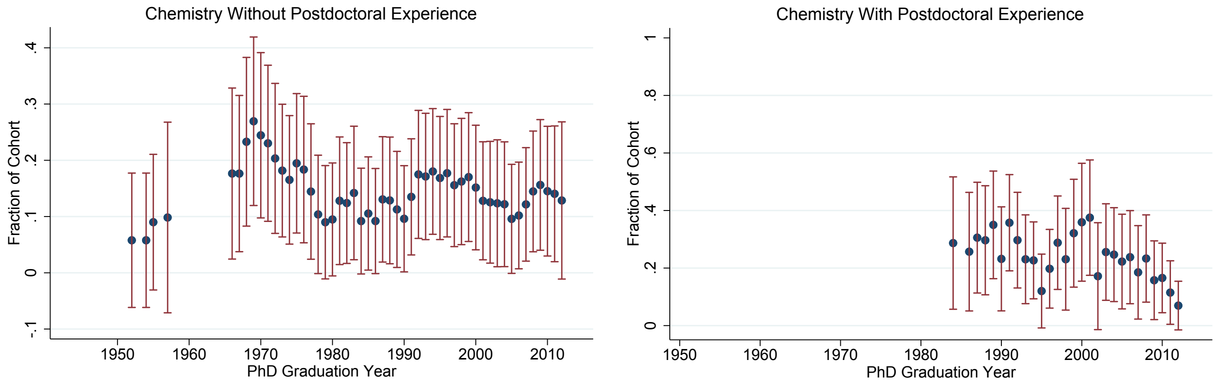

B:

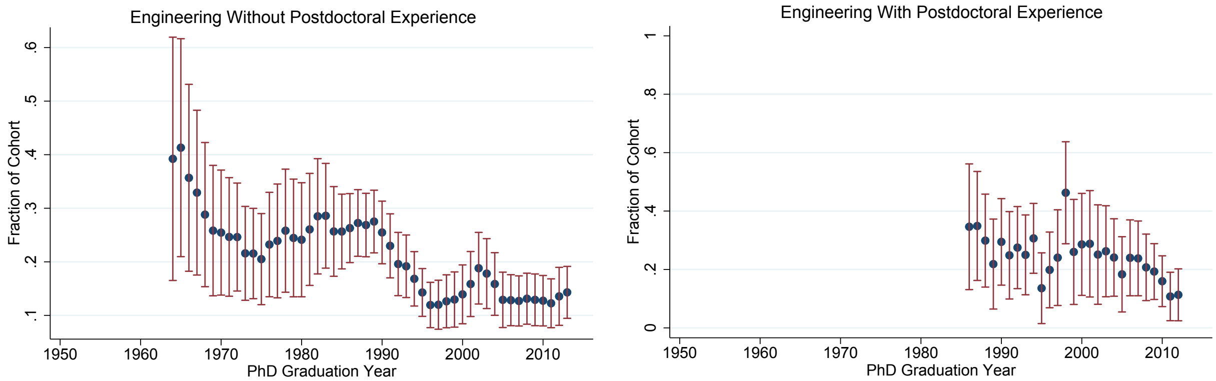

C:

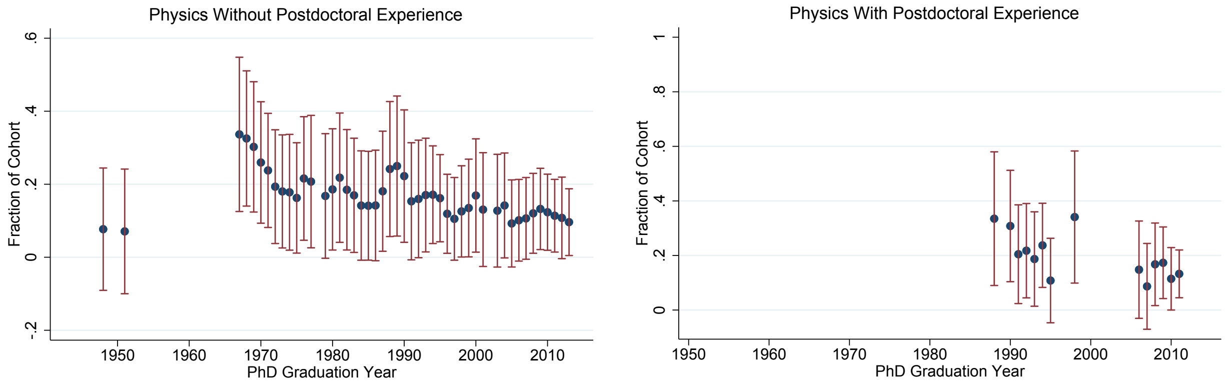

S2 Fig. 3. Fraction Observed in an Academic Tenure-Track Position Without and With Postdoctoral Experience by Ph.D. Cohort for Additional Fields. These graphs give the three-year moving 95% confidence intervals for the fraction of each Ph.D. cohort observed in an academic tenure-track position (left) within two years of their Ph.D. graduation without any postdoctoral experience and (right) within two years after their last postdoctoral appointment for A: Chemistry, B: Engineering, and C: Physics. N ranges from {50, 149} for chemistry, {50, 824} for engineering, and {50, 139} for physics.

A:

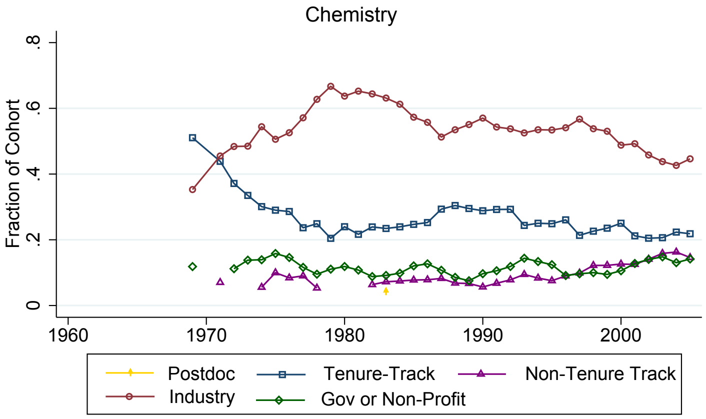

B:

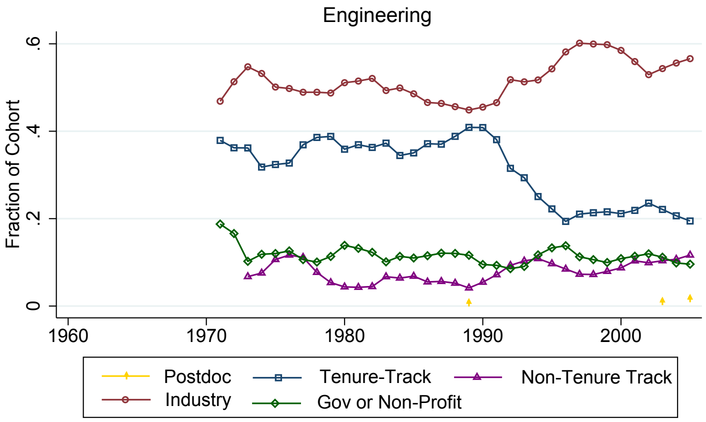

C:

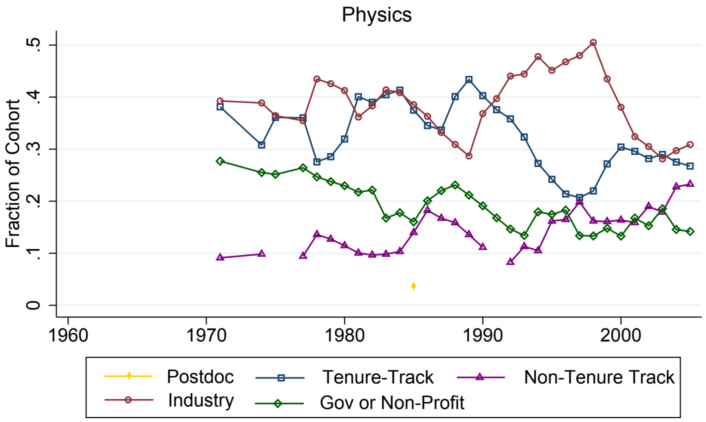

S2 Fig. 4. Job Distributions Ten Years Post-Ph.D. Graduation for Additional Fields. These graphs give the three-year moving fraction of each Ph.D. cohort working ten years post-Ph.D. graduation in each job type for A: Chemistry, B: Engineering, and C: Physics. Individuals who are not working or do not have data ten years post-Ph.D. are not included. N ranges from {53, 166} for chemistry, {66, 468} for engineering, and {54, 111} for physics.

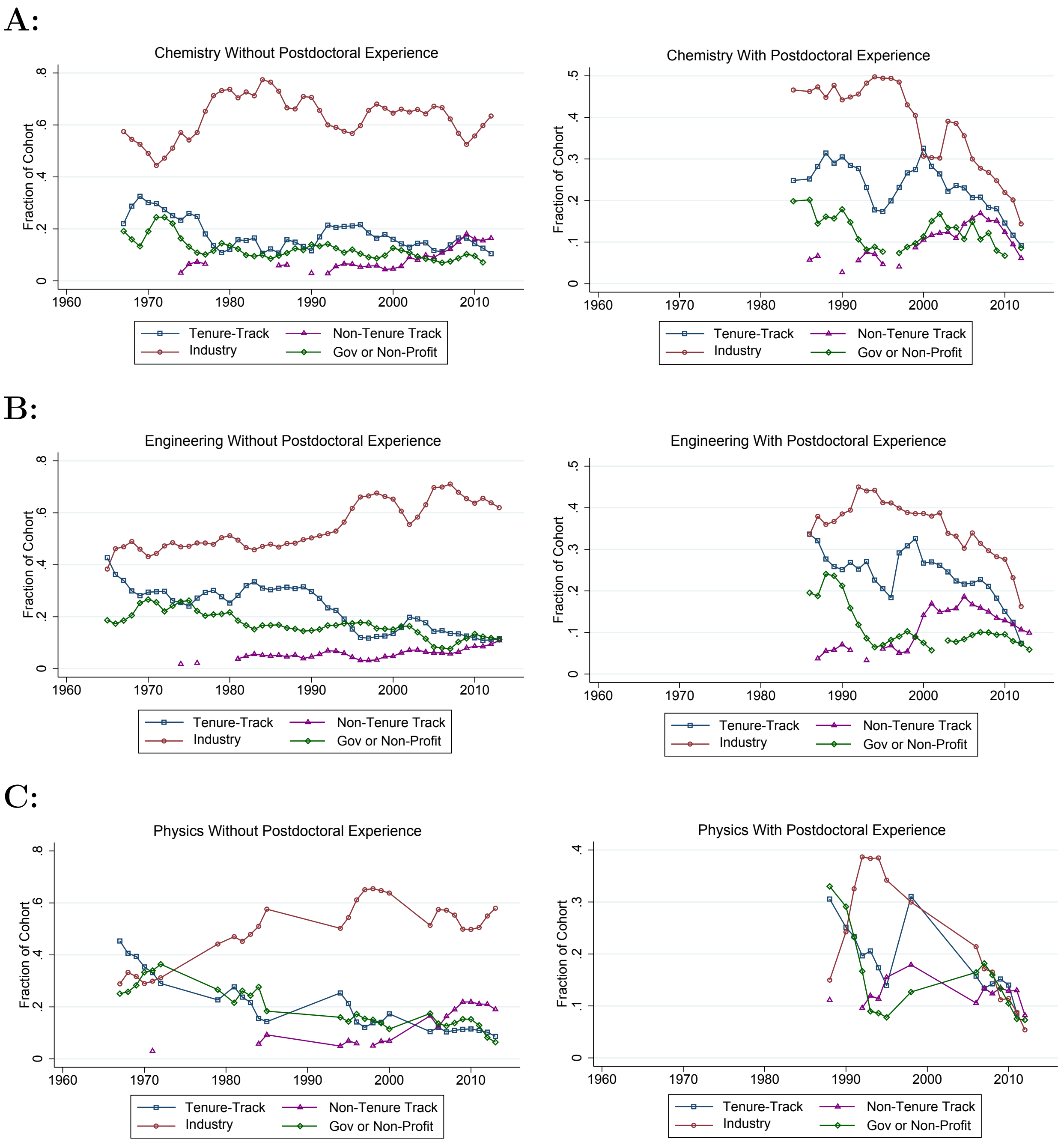

S2 Fig. 5. Distribution of Non-Postdoctoral Job Transitions Without and With Postdoctoral Experience by Ph.D. Cohort for Additional Fields. These graphs give the three-year moving distribution of each Ph.D. cohort who transition into each non-postdoc job type (left) within two years of their Ph.D. graduation with no postdoctoral experience and (right) within two years after their last postdoctoral appointment for A: Chemistry, B: Engineering, and C: Physics. N ranges from {50, 130} for chemistry, {56, 729} for engineering, and {50, 139} for physics.

A:

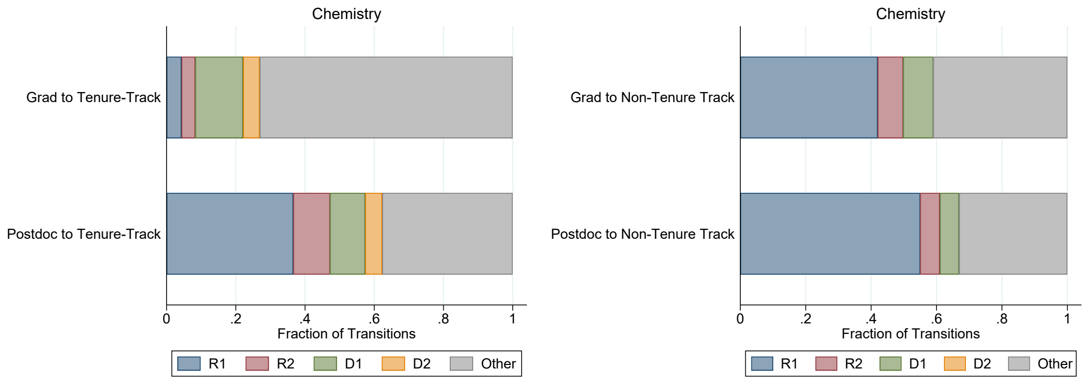

B:

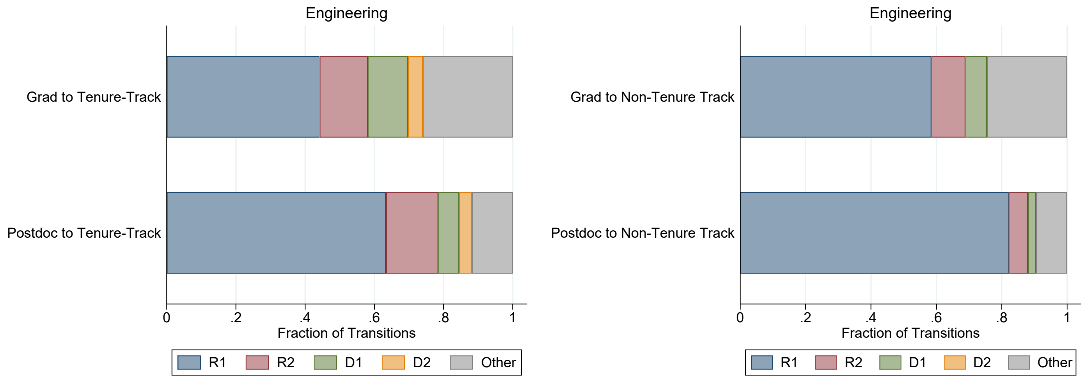

C:

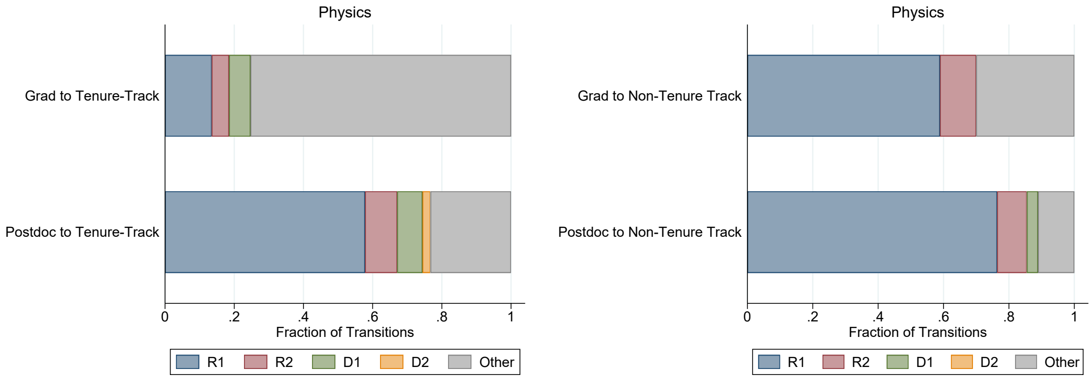

S2 Fig. 6. Institutional Carnegie Classifications for Academic Transitions Without and With Postdoctoral Experience for Additional Fields. These graphs give the distribution of known Institutional Carnegie Classifications for academic transitions to (left) tenure-track positions and (right) non-tenure track positions for A: Chemistry, B: Engineering, and C: Physics. "Grad" transitions are within two years of their Ph.D. graduation with no postdoctoral experience. "Postdoc" transitions are within two years after their last postdoctoral appointment. Institutional prestige is highest among R1 ("very high research") institutions, followed by R2 ("high research"), D1 ("doctoral I"), D2 ("doctoral II"), and Other (no available classification). N ranges from {111, 820} for chemistry, {256, 1250} for engineering, and {115, 595} for physics.

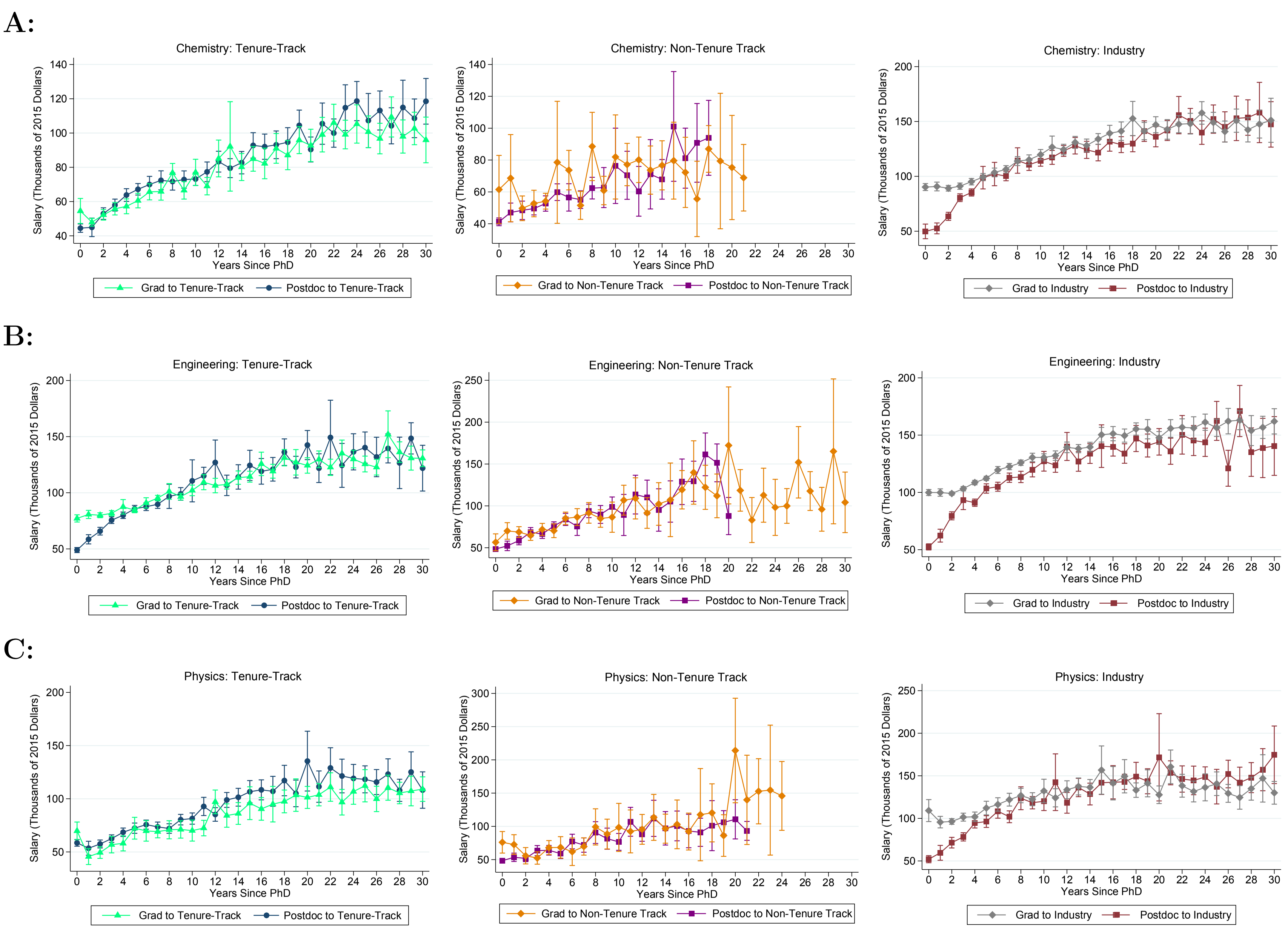

S2 Fig. 7. Average Salary in Each Year Since Ph.D. Graduation by Postdoctoral Path for Additional Fields. These graphs give the average salary in (left) tenure-track jobs, (middle) non-tenure track jobs, and (right) industry jobs over the first thirty years after Ph.D. graduation without and with any postdoctoral experience for A: Chemistry, B: Engineering, and C: Physics. N ranges from {51, 2652} for chemistry, {58, 9495} for engineering, and {50, 1340} for physics.
